# Supplementary material for: Physician Perspectives on Malnutrition Screening, Diagnosis, and Management: A Qualitative Analysis
Source: Nutrients. 2024 Jul 11;16(14):2215. doi: 10.3390/nu16142215 (PMC11279970; doi:10.3390/nu16142215)
Supplement: Supplementary file 1 [file nutrients-16-02215-s001.zip › nutrients-3078709-supplementary.pdf]

Table S1: Interview Questions Posed To Participants Based in Nova Scotia

1. How is nutrition involved in the recovery of inpatients under your care? How important do you think it is for you to know or determine the nutritional status of hospitalized patients?
2. How are malnourished patients identified on your unit (i.e. low albumin, subjective global assessment)? What steps do you take when you have a patient that is malnourished?
3. Who would you talk to regarding nutrition questions for your hospitalized patients?
4. What do you see as your role in managing malnutrition in hospitalized patients including on discharges and transitions to the community?
5. How would you follow-up on a patient's nutritional status?
6. Are you familiar with the Subjective Global Assessment (SGA) tool used to determine a patient's nutritional status (Appendix E)? What are your thoughts on its use?
7. The INPAC pathway has been used in hospitals to promote the detection, prevention, and treatment of malnutrition. Where do you think the physician comes in with the care of patients who may be malnourished?
8. What tools and resources are needed to manage malnourished patients (i.e. periodic weights, treating symptoms contributing to poor intake, initiating investigations when needed, involving/referring to other healthcare professionals, prescribing oral supplements)?
9. What would you like to know more about preventing, detecting, or treating malnutrition?
10. What are the barriers that prevent physicians from being able to competently and confidently manage malnutrition concerns in hospitalized patients?
11. What resources would help you and your team with implementing nutrition care?

Table S2: Interview Questions Posed to Participants Based in Saskatchewan and Alberta (Changes identified in italics).

1. How is nutrition involved in the recovery of inpatients under your care? How important do you think it is for you to know or determine the nutritional status of hospitalized patients?
2. How are malnourished patients identified on your unit (i.e. low albumin, subjective global assessment)? What steps do you take when you have a patient that is malnourished?
3. *Which healthcare professionals are most knowledgeable about nutrition related patient care? How frequently do you discuss nutrition related care with that healthcare professional?*
4. *What do you see as your role in managing malnutrition in the patients under your care? Who do you see as the leaders in nutrition-related care for patients?*
5. *Do you schedule follow-up for patients to specifically address nutrition status? If so, what clinical or biochemical parameters do you use to view improvement or decline?*
6. *Have you ever heard about the Subjective Global Assessment (SGA)? (If yes), Have you ever used the SGA in clinical practice? (If yes) How often do you use SGA? Do you think it is useful in daily practice? Are you able to name some of the components of the SGA from memory?*
7. *What in your opinion are the top three clinical or biochemical markers that indicated that a patient is malnourished?*
8. *Are you aware of any clinical tool or pathway that could assist in detection, prevention, and/or treatment in malnourished patients? If so, which ones?*
9. What tools and resources are needed to manage malnourished patients (i.e. periodic weights, treating symptoms contributing to poor intake, initiating investigations when needed, involving/referring to other healthcare professionals, prescribing oral supplements)?
10. What would you like to know more about preventing, detecting, or treating malnutrition?
11. What are the barriers that prevent physicians from being able to competently and confidently manage malnutrition concerns in hospitalized patients?
12. What resources would help you and your team with implementing nutrition care?
